# The Morphology and Assembly of Respiratory Syncytial Virus Revealed by Cryo-Electron Tomography

**DOI:** 10.3390/v10080446

**Published:** 2018-08-20

**Authors:** Zunlong Ke, Rebecca S. Dillard, Tatiana Chirkova, Fredrick Leon, Christopher C. Stobart, Cheri M. Hampton, Joshua D. Strauss, Devi Rajan, Christina A. Rostad, Jeannette V. Taylor, Hong Yi, Raven Shah, Mengtian Jin, Tina V. Hartert, R. Stokes Peebles, Barney S. Graham, Martin L. Moore, Larry J. Anderson, Elizabeth R. Wright

**Affiliations:** 1 Division of Pediatric Infectious Diseases, Emory University School of Medicine, Children’s Healthcare of Atlanta, Atlanta, GA 30322, USA; zunlongke@gatech.edu (Z.K.); rebecca.s.dillard@emory.edu (R.S.D.); tania.chirkova@emory.edu (T.C.); fredrick.leon@emory.edu (F.L.); cstobart@butler.edu (C.C.S.); cheri.hampton.ctr@us.af.mil (C.M.H.); joshua.d.strauss@emory.edu (J.D.S.); drajan@emory.edu (D.R.); christina.rostad@emory.edu (C.A.R.); raven.shah@emory.edu (R.S.); mengtian.jin@emory.edu (M.J.); mlmoor2@emory.edu (M.L.M.); larry.anderson@emory.edu (L.J.A.); 2 School of Biological Sciences, Georgia Institute of Technology, Atlanta, GA 30332, USA; 3 Robert P. Apkarian Integrated Electron Microscopy Core, Emory University, Atlanta, GA 30322, USA; jvtaylo@emory.edu (J.V.T.); hyi@emory.edu (H.Y.); 4 Division of Allergy, Pulmonary, and Critical Care Medicine, Vanderbilt University School of Medicine, Nashville, TN 37232, USA; tina.hartert@Vanderbilt.Edu (T.V.H.); stokes.peebles@Vanderbilt.Edu (R.S.P.J.); 5 Vaccine Research Center, National Institute of Allergy and Infectious Diseases, National Institutes of Health, Bethesda, MD 20892, USA; bgraham@mail.nih.gov

**Keywords:** human respiratory syncytial virus (RSV), cryo-electron tomography (cryo-ET), cryo-electron microscopy (cryo-EM), enveloped virus, virus assembly, viral morphogenesis

## Abstract

Human respiratory syncytial virus (RSV) is the leading cause of lower respiratory tract disease in young children. With repeat infections throughout life, it can also cause substantial disease in the elderly and in adults with compromised cardiac, pulmonary and immune systems. RSV is a pleomorphic enveloped RNA virus in the *Pneumoviridae* family. Recently, the three-dimensional (3D) structure of purified RSV particles has been elucidated, revealing three distinct morphological categories: spherical, asymmetric, and filamentous. However, the native 3D structure of RSV particles associated with or released from infected cells has yet to be investigated. In this study, we have established an optimized system for studying RSV structure by imaging RSV-infected cells on transmission electron microscopy (TEM) grids by cryo-electron tomography (cryo-ET). Our results demonstrate that RSV is filamentous across several virus strains and cell lines by cryo-ET, cryo-immuno EM, and thin section TEM techniques. The viral filament length varies from 0.5 to 12 μm and the average filament diameter is approximately 130 nm. Taking advantage of the whole cell tomography technique, we have resolved various stages of RSV assembly. Collectively, our results can facilitate the understanding of viral morphogenesis in RSV and other pleomorphic enveloped viruses.

## 1. Introduction

Human respiratory syncytial virus (RSV) is the leading cause of lower respiratory tract disease in young children including bronchiolitis and pneumonia. With repeat infections throughout life, RSV causes substantial disease in older children and adults with compromised cardiac, pulmonary and immune systems, as well as the elderly. In severe cases, complications due to the disease can lead to death [1,2,3]. With significant morbidity and mortality in the United States and disproportionately high-levels worldwide, it has been a high priority for vaccine development for over 50 years but no licensed vaccine is yet available [4,5].

RSV is an enveloped, negative sense, single-stranded RNA virus in the family of *Pneumoviridae* [1,6]. The ~15.2 kb genome of RSV contains 10 open reading frames, encoding nine structural proteins and two non-structural proteins. The attachment glycoprotein (G), fusion glycoprotein (F), and the small hydrophobic protein (SH) are anchored in the viral membrane with the majority of the protein present on the exterior of the membrane; the matrix protein (M) lines the interior of the viral membrane. The viral genomic RNA is encapsidated in the ribonucleoprotein complex (RNP) that is composed of the nucleoprotein (N), phosphoprotein (P), and the RNA-dependent RNA polymerase (RdRp, L) [1]. This nucleoprotein-RNA complex forms a helical assembly and serves as a template for virus replication [7,8]. The M2 gene encodes two proteins, M2-1 and M2-2. M2-1 is an essential transcription anti-terminator that binds to RNA and is important for the synthesis of the full-length mRNAs [9,10]. Structurally, M2-1 forms a tetramer. It also functions as a linker protein between M and the RNP and is required for regulating RSV structural organization [11,12,13,14]. The two non-structural proteins, NS1 and NS2, encoded by the two promoter-proximal genes, have been suggested to facilitate virus growth by regulating type I interferon (IFN) activation and response pathways, but their exact targets are yet to be characterized [15,16,17].

The two major antigens, F and G, protrude from the surface of the viral membrane and are the only two proteins that are targeted by neutralizing antibodies [18]. While G has an epitope in the central conserved domain with neutralization-sensitive properties [18,19,20], F is a more potent and cross-protective candidate for RSV vaccine design and structure-directed drug development [4,18,21,22,23,24]. F is a 574-amino acid class I fusion protein that forms a trimeric structure with a thermodynamically metastable prefusion state, numerous intermediate conformational states, and a stable postfusion state [25,26]. During the viral fusion process, the trimeric metastable prefusion form of F rearranges into the irreversible 6-helix bundle postfusion form, which initiates the fusion pore formation between the viral membrane and the host cell plasma membrane [27]. Due to the essential role of prefusion-F in the virus entry process, maintaining F in this conformational state is required to elicit a high-level host immune response. Studies have shown that formalin-inactivated RSV (FI-RSV) leads to vaccine enhanced respiratory disease [28,29], and this can likely be attributed to the fact that prefusion-F is nearly absent on the surface of FI-RSV [30]. Thus, prefusion-F based immunogens are better candidates, as demonstrated in recent studies on platforms of both live-attenuated RSV [23,24] and subunit vaccines [22,31].

It has been suggested that M is the driving force for the assembly of RSV [32,33,34,35] and other related paramyxoviruses [36,37]. A recent study by the Oomens group found that an RSV M-null mutant exhibited failed RSV viral filament elongation, indicating the role of the RSV M protein in driving filamentous particle formation [33]. RSV M forms a dimer and mutations at the M dimer interface prevent assembly of both virus-like particles (VLPs) and viral filaments [38]. Bajorek et al. demonstrated that residue Thr205 of the RSV M protein is responsible for the higher-order oligomerization of RSV M, and mutations of Thr205 result in shortened RSV filament formation. Thus, the higher-order oligomerization of RSV M plays a role in RSV filament elongation [39]. Although M is the impetus for filament formation, interactions between M and the F cytoplasmic tail (CT) have also been suggested to be essential for RSV viral filament formation [40]. Our recent cryo-ET study of measles virus assembly highlighted the ordered structural relationship between F and M. We resolved on measles virus particles and at sites of assembly that F and M form a “double-layered” lattice on the viral particles [36], which indicates the structural and functional interactions between M and the CT domain of F. Fluorescence microscopy combined with scanning electron microscopy (SEM) has shown that RSV F can initiate short filament formation in the absence of M [33].

There are two potential pathways for RSV filament assembly [41]. One possibility is that virus assembly and maturation occur at the plasma membrane, similar to several closely related paramyxoviruses [41,42]. An alternative route is that some steps of virus assembly happen within the cytoplasm before the complexes reach the plasma membrane [41]. Recently, the Santangelo group proposed a model for RSV filament assembly. Their fluorescence microscopy results imply that RSV filaments loaded with RSV proteins and genomic RNA are formed in the cytoplasm prior to reaching the plasma membrane [43], suggesting the alternate pathway. However, to date, there have not been cryo-electron microscopy (cryo-EM) or correlative light and electron microscopy (CLEM) based studies of RSV-infected cells which would further clarify the stages of and structures formed during RSV assembly.

In this study, we demonstrate that RSV particles are filamentous when produced from virus-infected human-derived cell lines and virus-infected polarized normal human bronchial epithelial (NHBE) cells. Our quantitative analysis of the cryo-EM/ET data shows that the average length of the filaments is around 1.5 µm with an average diameter of ~130 nm. The architecture of RSV is further validated by immunogold labeling of both frozen-hydrated RSV-infected cell lines and fixed RSV-infected polarized NHBE cells. In addition, we resolve that the RSV F glycoproteins are in the metastable prefusion state on the filamentous viral particles. Furthermore, the RSV assembly steps captured by cryo-ET provide a better understanding for the formation of the filamentous RSV particles. Our results suggest that M regulates RSV morphology since the M layer associates with the viral membrane in filamentous particles where the viral membrane is straight. To our knowledge, we are the first to reveal RSV assembly steps at the plasma membrane in the native state at macromolecular resolution using cryo-ET. This will shed light on the establishment of the relationship between RSV morphology, the conformational states of the surface glycoproteins, and viral infectivity, thus contributing to vaccine development and structure-directed antiviral drug design.

## 2. Materials and Methods

### 2.1. Cell Culture

HEp-2 (ATCC CCL-23), Vero (ATCC CCL-81), HeLa (ATCC CCL-2), A549 (ATCC CCL-185), and MRC-5 (ATCC CCL-171) cells were cultured and maintained in DMEM complete medium, supplemented with 10% fetal bovine serum (FBS), 1 µg/mL penicillin, streptomycin, and amphotericin B (PSA) antibiotics. BEAS-2B cells (ATCC CRL-9609) were cultured and maintained in RPMI-1640 complete medium supplemented with 10% FBS and 1 µg/mL PSA [44,45].

Polarized normal human bronchial epithelial (NHBE) cells were kindly provided by Dr. Calvin Cotton (Case Western Reserve University) and cultured as described previously [46,47]. Polarized NHBE cells cultured at the air-liquid interface retain similar morphological and physiological properties as the human respiratory epithelium, including its susceptibility to RSV [48]. Briefly, cells were grown on 12 mm Costar Transwell inserts (Corning Inc., Corning, NY, USA) until confluent, then transferred to the air-liquid interface where cells were maintained in primary airway medium containing 2% Ultroser G (Pall Biosepra, SA, Cergy-Sainte-Christophe, France) until differentiation was evident and resistance measurements were >500 ohms.

### 2.2. Viruses and Infection

RSV strains used in this study are A2 strain, rA2-mK^+^ strain, and TN strain. A2 is a lab adapted wild-type strain. Recombinant virus strain rA2-mK^+^ was generated using a reverse genetics system as previously described [49]. This recombinant virus is an A2 strain with a far-red fluorescent reporter gene (monomeric Katushka 2 (mK^+^)) incorporated in the first position of the RSV antigenomic cDNA. Incorporation of the mK^+^ does not affect RSV growth dynamics in vitro or in mice [49]. The fluorescent reporter is used for the fluorescent focal unit (FFU) assay for virus titration. The TN strain (A/TN/12/11-19) was isolated from an infant with acute RSV infection enrolled in a research study at Vanderbilt University and kept at low passage prior to investigation in this study. Viruses were propagated in HEp-2 and/or Vero cells and stored at −80 °C.

For cryo-EM sample optimization studies, BEAS-2B cells were seeded at 1.6 × 10^5^ cells/well in 6-well plates, infected with the rA2-mK^+^ strain at various multiplicities of infection (M.O.I.), and incubated for 24 h at 37 °C and 5% CO_2_. Cell supernatants were collected for real time RT-PCR to determine viral RNA as described below or used for virus titration in Vero cells to determine virus replication by FFU assay as described below. Cells were collected using Trypsin-EDTA and assessed by flow cytometry for the percentage of RSV-positive cells, also described below. For the early RSV replication assay, BEAS-2B cells were inoculated with rA2-mK^+^ at a M.O.I. of 10, and total cells were harvested every 6 h post infection (h.p.i.) up to 36 h.p.i. Viral titer was determined by FFU assays of infected Vero cells.

For cryo-EM/ET infection studies, cells (HeLa, A549, MRC-5, and BEAS-2B) were grown on gold Quantifoil R 2/1 grids (Quantifoil, Großlöbichau, Germany) in MatTek dishes (MatTek Corp., Ashland, MA, USA), inoculated with RSV strains A2 or TN at a M.O.I. of 10, incubated for 24 h, and processed for EM sample preparation and imaging as described [24,36,44,45].

For infection of the polarized human airway epithelial cells, NHBE cells were inoculated with RSV TN strain at a M.O.I. of 2 for 2 h, the viral inoculum was aspirated, and cells were incubated for 6 days at 37 °C and 5% CO_2_. After incubation, cells were processed for TEM imaging as described below. 

### 2.3. Virus Titration by Fluorescent Focal Unit (FFU) Assay

Virus titration was carried out by infecting Vero cells with serially diluted virus stocks. The cell seeding density was 2 × 10^4^ cells/well in 96-well plates. Virus attachment was facilitated by spinoculation at 3000 rpm at 4 °C for 30 min. The infected cells were semi-fixed with 0.75% methylcellulose DMEM complete medium. The fluorescent focal units (FFU/mL) were determined 48 h.p.i. using the mK^+^ fluorescent signal.

### 2.4. One-Step Growth Curve

BEAS-2B cells were seeded in 6-well plates with 1.6 × 10^5^ cells/well and incubated overnight. Cells were then infected with the rA2-mK^+^ strain at a M.O.I. of 10, followed by incubation with rocking at room temperature for one hour. After incubation, infected cells were washed twice with DPBS (with Ca^2+^ and Mg^2+^) and supplemented with fresh medium followed by incubation at 37 °C and 5% CO_2_. Every 6 h.p.i., infected cells were harvested and collected, starting at 1 h through 36 h. Samples were vortexed for 30 s prior to flash freezing in liquid nitrogen and stored at −80 °C until virus titration.

### 2.5. Detection of RSV-Infected Cells by Flow Cytometry

Cells harvested from plates as described above were washed with PBS and re-suspended in 1% formaldehyde in PBS and stored at 4 °C. Flow cytometry analysis was performed using BD LSR-II (BD BioSciences, Franklin Lakes, NJ, USA) and FlowJo software (Tree Star, Inc., Ashland, OR, USA). The percentage of RSV-positive cells was determined by the presence of mK^+^ signal. Uninfected cells were used as a negative control. 

### 2.6. RSV RNA Detection by Real Time RT-PCR Assay

The amount of viral RNA was determined by real time RT-PCR. Supernatant and cell pellet were collected at 24 h.p.i. The collected supernatant was briefly centrifuged to remove cell debris. RNA was extracted from the supernatant and cells using a Qiagen RNeasy mini kit according to the manufacturer’s instructions. RSV RNA was assayed by a real-time RT-PCR using AgPath-ID™ One-Step RT-PCR Reagents and the Applied Biosystems 7500 Fast Real-Time PCR System (Life Technologies Corporation, Carlsbad, CA, USA) as previously described [50]. The primers and probes for the RSV matrix (M) gene (forward primer, 5′-GGC AAA TAT GGA AAC ATA CGT GAA-3′; reverse primer, 5′-TCT TTT TCT AGG ACA TTG TAY TGA ACA G-3′; probe, 5′-6-carboxyfluorescein (FAM)-TGT CCG TCT TCT ACG CCC TCG TC-black hole quencher 1 (BHQ-1)-3′) were obtained from Integrated DNA Technologies (IDT) (Coralville, IA, USA) [50]. The cycle threshold (C_T_) values, the number of cycles required to exceed the background level, were calculated. Inverse C_T_ values (1/C_T_) were used to express the relative amount of RNA extracted from the cells or supernatant of the infected cells [20].

### 2.7. RSV Infection in the Presence of the Fusion Inhibitor BMS-433771

For cryo-EM/ET infection studies, HeLa cells were grown on gold Quantifoil R 2/1 grids (Quantifoil, Großlöbichau, Germany) and inoculated with RSV A2-mK^+^ at a M.O.I. of 10. The infected cells were rocked for 1 h at room temperature to facilitate virus binding and were then washed twice with PBS to remove the unbound viral particles. New medium containing 600 nM fusion inhibitor (BMS-433771) [51,52] was added to prevent viral entry and fusion events, and the infected cells with fusion inhibitor were allowed to incubate for 24 h. The samples were processed for cryo-EM/ET imaging as described below.

### 2.8. RSV Morphology Characterization

Only released, complete viral particles were quantified and measured. Particle length and diameter were both quantified in IMOD using the *imodinfo* command by creating a model file [53]. The filament length measurements were made by adding open contours in IMOD along the viral filament. The total length is the sum of all the segments. Diameter was measured between the two “sides” of the virus particles, perpendicular to membrane. The reported diameter is the average of measurements taken every 500 nm along the particle, with a minimum of three and maximum of ten measurements per particle. For the spherical particles, we performed similar analysis by taking four measurements of the diameter and obtaining an average diameter. Some virus-infected cells produce more spherical viruses (~30 particles) while others produce fewer (~ 5–10 particles). Due to stochastic sampling and the low percentage (~5%) of spherical particles in the population, we did not perform quantitative characterization of these viruses.

### 2.9. Thin Sectioning TEM of RSV-Infected Polarized NHBE Cells

Immunogold labeling and thin section TEM procedure details were described previously [44]. Briefly, at 6 days post infection (d.p.i.) at a M.O.I. of 0.05, the NHBE cells were washed 3 times with PBS to remove mucus, which can potentially prevent antibody binding to the surface of viral particles. Primary antibody (motavizumab, 5 µg/mL) was applied and the cells were incubated for 1.5 h, followed by washing in warmed medium 4 times before incubation with the secondary antibody (goat-anti-human conjugated to 6 nm gold markers) for 1.5 h. Cells were quickly washed with 2.5% glutaraldehyde in 0.1 M phosphate buffer and fixed overnight at 4 °C in 2.5% glutaraldehyde. Cells were then post-fixed with 0.1% osmium tetroxide in 0.1 M phosphate buffer (pH 7.4) for an hour, followed by dehydration in graded ethanol (25%, 50%, 75%, 95%, and 100%). Infected cells were then infiltrated, embedded, and polymerized in Eponate 12 resin (Ted Pella Inc., Redding, CA, USA). Ultrathin sections of RSV-infected NHBE cells were cut using a Leica Ultracut S ultramicrotome at ~70 nm thickness. Sections were then stained with 5% uranyl acetate and 2% lead citrate and imaged using a JEOL JEM-1400 TEM (JEOL Ltd., Tokyo, Japan) with Gatan US1000 2k × 2k CCD camera (Gatan, Pleasanton, CA, USA).

### 2.10. Cryo-EM/ET Sample Preparation, Data Collection, and Image Processing

Grids with infected cells (as described above) were plunge frozen using a Gatan CryoPlunge 3 system (Gatan, Plesanton, CA, USA). 10 nm gold fiducials were applied to the grids prior to freezing. Polygon montages were collected using US4000 4k × 4k CCD camera (Gatan, Pleasanton, CA, USA), with an effective pixel size of 11.80 Å (10,000 × nominal magnification) on the specimen level [36]. Polygon montages were blended using *extractpieces* and *sloppyblend.com* script in IMOD, and the blended montage was Fourier filtered in IMOD for better visualization. Bi-directional tilt series were collected semi-automatically using the SerialEM package [54,55], with a tilt range of −65 to +65 degrees at 2-degree tilt increment. A cumulative electron dose of 120 e^−^/Å^2^ to 140 e^−^/Å^2^ was used. Images were recorded using a Direct Electron DE-20 camera (Direct Electron, LP, San Diego, CA, USA) at 10,000× nominal magnification (6.14 Å/pixel on the level of specimen) with -6 µm defocus using a JEOL JEM-2200FS TEM (JEOL Ltd., Tokyo, Japan). In order to correct the motion induced by beam exposure, all frames were motion corrected using python script (DE_process_frames-2.8.1.py) provided by Direct Electron, LP. Tilt series were reconstructed from motion-corrected images with IMOD software [53]. Linear density profiles were plotted from the Gaussian low-pass filtered (80 Å) tomographic slices of the reconstructed tomograms with the Fiji software using Analyze/Plot Profile [56]. In order to represent the overall structural organization, the linear density profiles were prepared from multiple regions where the membrane, M, M2-1 and RNP were oriented together. The final plots were the averaged measurements from a number of regions. In order to visualize the 3D volumes, manual segmentation was performed using Amira software (FEI, Visualization Sciences Group, Hillsboro, OR, USA). Model fitting of the two states of the F glycoprotein into the isosurface rendering of the tomographic data was done manually in Chimera software [57]. Reconstructed tomograms were first Gaussian low-pass filtered to 80 Å using *e2proc3d.py* in EMAN2 [58], then a small region was extracted and contrast inverted prior to loading the map into Chimera. Prefusion F (PDB ID: 4JHW) [26] and postfusion F (PDB ID: 3RRT) [27] were manually modeled into the F glycoproteins on the filamentous and spherical particles, respectively.

### 2.11. Graphs and Statistical Analysis

All graphs and statistical analyses were done in Graph Pad Prism version 6.0 (GraphPad Software, La Jolla, CA, USA).

## 3. Results

### 3.1. Optimization of the Conditions for Cryo-EM/ET Sample Preparation

**Figure 1:**
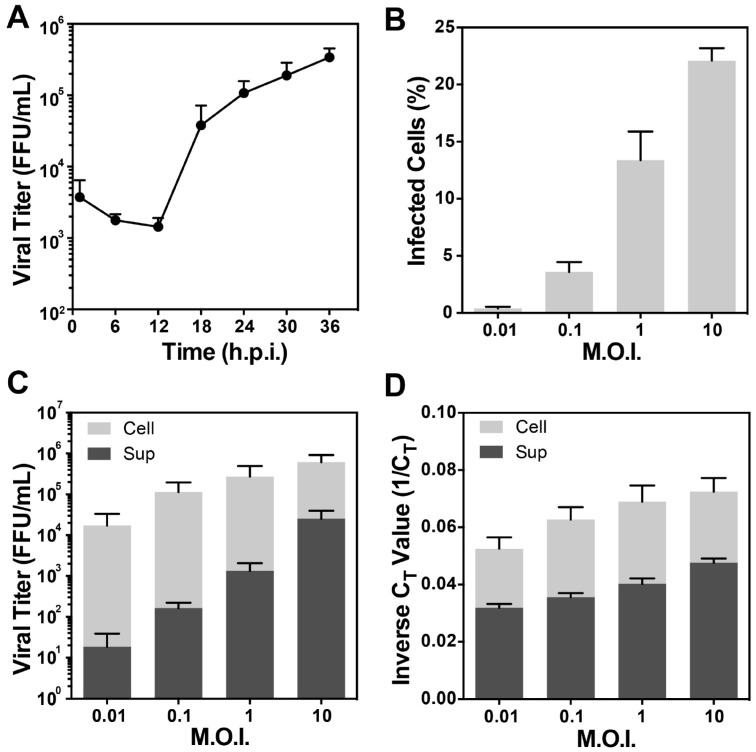
Assessment of sample preparation conditions for cryo-EM data collection. (**A**) One-step growth curve of RSV A2 in BEAS-2B cells over a period of 36 h at a M.O.I. of 10. Infected cells were harvested at indicated time points and titrated in Vero cells. (**B**–**D**) Flow cytometry, viral titer, and RNA quantification analysis of RSV-infected BEAS-2B cells, at indicated M.O.I.s after 24 h.p.i. These data indicate that at a M.O.I. of 10, infectious particles are produced at 24 h.p.i., suggesting that the particles being imaged by cryo-EM are infectious. (**B**) Relative ratio of cells infected at indicated M.O.I. by flow cytometry using mK^+^ signal, represented by the ratio of infected cells over the total cells. (**C**) Viral titer at 24 h.p.i. determined by FFU using mK^+^ signal. (**D**) Relative amount of viral RNA from infected cells determined by real time RT-PCR represented by inverse C_T_ values (1/C_T_). C_T_ value is the number of cycles needed to pass the background level. For all 4 experiments, error bars represent the standard deviation of 3 independent experiments.

**Figure 2:**
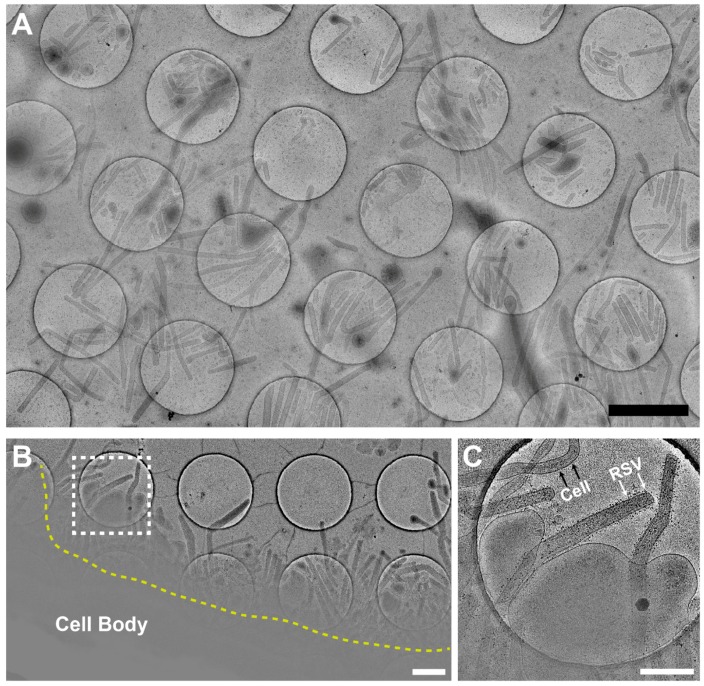
Representative montages showing 2D projections of RSV-infected cells. (**A**) Representative montages showing released viruses from RSV-infected cells. Montage view of released viral particles captured from RSV A2 infected A549 cells. Sample was prepared as described in the method section. RSV morphology varies, with the overall structure being filamentous. Filaments may be straight, bent, or branched. (**B**) Montage view of the assembly site from RSV-infected cells with the F glycoprotein immunogold labeled. The region imaged is an active assembly site, with RSV filaments extending from the cell plasma membrane (dashed yellow line). (**C**) Zoomed-in view of the boxed region in B. Note the presence of 6-nm immunogold on the RSV filaments (white arrows), and the absence of gold particles on cell extension (black arrows). Scale bars are 2 µm (**A**), 1 µm (**B**), and 500 nm (**C**).

In order to achieve an appropriate balance between the infectivity and the quantity of RSV produced from the infected cells and low levels of cell confluency (30–50%), we tested the infection conditions at different combinations of time points and M.O.I.s [24,44,45,59]. One-step growth curves showed that at the 24-h time point the viral titer reached ~10^5^ FFU/mL even at very low cell confluency, indicating cells are actively producing infectious particles at this stage (Figure 1A). Flow cytometry results showed that more than 20% of the cells were infected at a M.O.I. of 10 (Figure 1B), and the titer of the cell-associated viruses (cell) reached ~10^6^ FFU/mL (Figure 1C). Titration and RNA quantification analyses of different M.O.I.s at 24 h.p.i. show that with an increased M.O.I., both the viral titer and the total viral genomic copy of the cell-associated viruses (cell) and released viruses in the supernatant (sup) increased proportionally (Figure 1C,D). Our results suggested that at a M.O.I. of 10 at 24 h.p.i., the cells actively produced infectious particles, indicating that the virus particles resulting from these conditions were infectious and biologically relevant (Figure 2). Filamentous particles have also been frequently observed by fluorescence light microscopy and cryo-EM under these conditions in multiple cell lines [45]. We thus elected to perform cryo-EM and cryo-ET experiments using a M.O.I. of 10 at 24 h.p.i.

### 3.2. RSV Is Filamentous Independent of Cell Line

**Figure 3:**
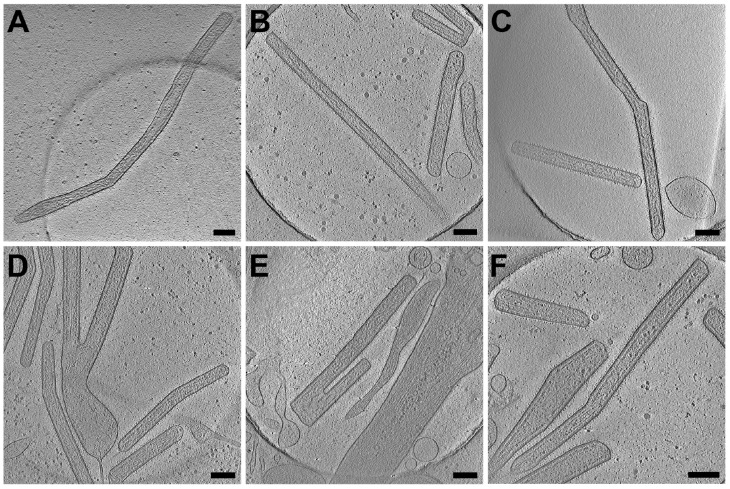
Cryo-ET of released RSV filaments from RSV-infected cells. (**A**–**F**) Cell lines including HeLa (**A**,**F**) and lung derived cell lines A549 (**B**), MRC-5 (**C**), and BEAS-2B (**D**,**E**), were infected with RSV A2 strain (**A**–**D**) or RSV TN strain (**E**,**F**). Among these samples, filamentous particles were consistently observed under frozen-hydrated conditions. Scale bars are 200 nm.

To determine whether RSV morphology is cell line or virus strain dependent, we performed cryo-EM and cryo-ET data collection on virus-infected cells (Figure 2 and Figure 3). We acquired montage images of ~2000 RSV particles from 2 virus strains and 4 cell lines. Our 2D imaging data confirmed what has previously been reported about the pleomorphic structure of RSV [11,12]. However, our examination of a large population of virus particles from multiple virus strains and cell lines allowed us to determine that the majority of RSV particles produced are filamentous (Figure 2 and Figure 3). We have captured straight, bent, and branched filaments (Figure 2). Using native cryo-immunogold labeling of the RSV F glycoprotein [44], we identified assembling and released filamentous RSV particles (Figure 2B,C). 3D cryo-ET reconstructions of the released virus particles indicate that the RSV A2 strain is filamentous from infected HeLa cells (Figure 3A). The morphology of the RSV A2 strain is not cell type specific, as demonstrated in three other human lung-derived cell lines, including human alveolar epithelial cell line (A549), human bronchial epithelial cell line (BEAS-2B), and human fetal lung fibroblasts (MRC-5) (Figure 3B–D). In order to investigate whether the phenotype is virus strain specific, we tested a low-passaged clinical isolate strain (A/TN/12/11-19, TN strain). Our results show that the morphology is consistently filamentous for the TN strain when propagated from both BEAS-2B and HeLa cells (Figure 3E,F).

### 3.3. RSV Morphology Quantification

**Figure 4:**
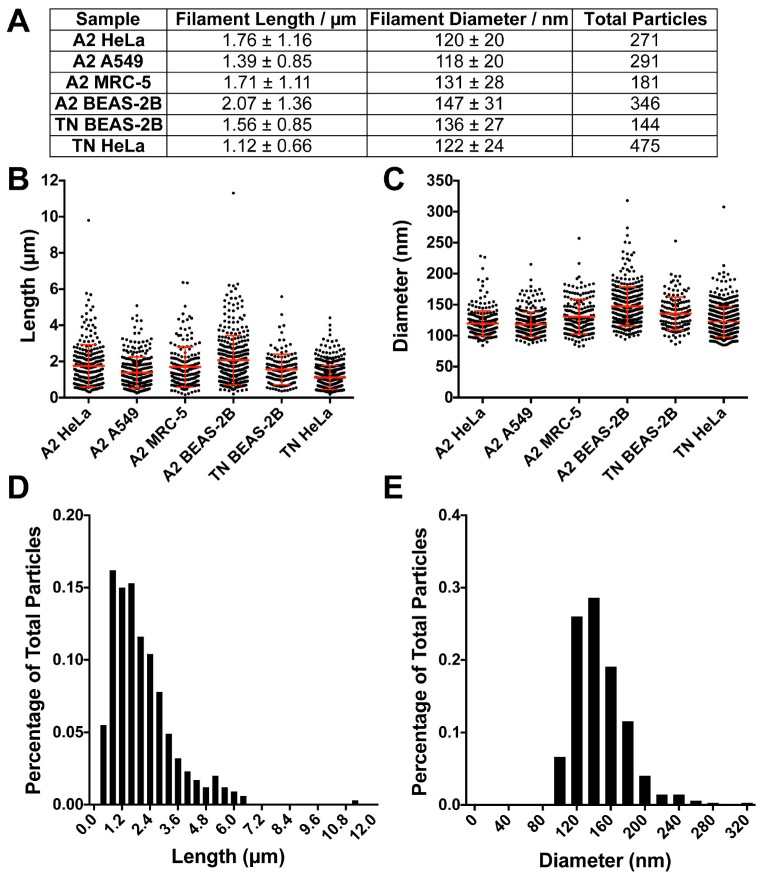
RSV morphology quantification in multiple virus strains and infected cell lines. (**A**) Overall quantification of RSV filament length and diameter. (**B**) RSV filament length distribution in indicated virus-infected cells. (**C**) RSV filament diameter distribution. Note, the mean diameter of RSV filaments is around ~130 nm. (**D**,**E**) Filament length (**D**) and diameter (**E**) distribution from A2-infected BEAS-2B cells.

We performed a quantitative analysis of the released viral particles to determine whether viral filament length or diameter varied in viruses produced from different cell types. First, we imaged newly released RSV particles from RSV-infected cells by cryo-EM (Figure 4). We measured the filament length and diameter in the IMOD program using the *imodinfo* command [53]. Among the 1800 particles quantified, 94% of them were filamentous. Of the filamentous particles, virus length varied from 0.5 to 12 µm, with an average of ~1.5 µm; the filament diameter was more consistent, with an average diameter of 130 nm for all the viruses in different cell lines, although it ranged from 100 nm to 250 nm (Figure 4A–C). The results are consistent with previous conventional EM studies that the filamentous particle size can be up to 10 µm in length and an average of ~200 nm in diameter [60,61]. The distribution of both filament length and diameter in all groups are similar to each other (Figure 4D,E). For the spherical particles, we performed a similar analysis by taking four measurements of the diameter and obtaining an average diameter of the spherical particles. However, due to stochastic sampling and low numbers of spherical particles in the population, there were virus-infected cells with more spherical viruses (~30 particles) while others had fewer (~5–10 particles). We found the spherical virus particles are morphologically similar to what has been previously reported [11,12], with the viruses maintaining an average diameter of ~300–400 nm. Based on the detailed structural analysis of the six types of virus-cell combinations, we conclude that the predominant morphology of RSV from infected cells is filamentous.

### 3.4. RSV Morphology Is Filamentous from RSV-Infected Polarized NHBE Cells

**Figure 5:**
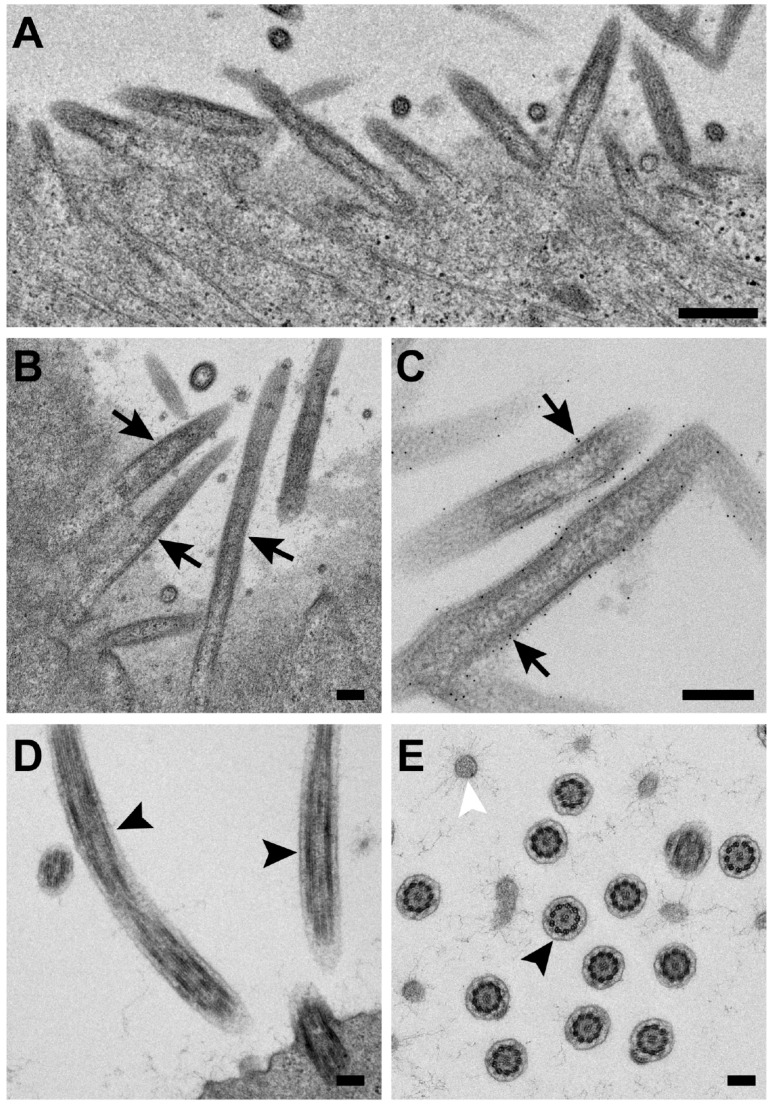
RSV morphology is filamentous from infected-NHBE cells. (**A**–**C**) Filamentous RSV particles were observed at virus assembly sites on infected cells. Black arrows indicate filamentous RSV. (**D**,**E**) Cellular filaments observed from the polarized NHBE cells, such as cilia (black arrowheads) and microvilli (white arrowhead). Scale bars are 200 nm.

To further assess RSV morphology in a physiologically relevant environment, we performed thin-section immunogold TEM to analyze RSV structure from RSV-infected NHBE cells. Due to the culturing conditions required for maintaining polarized NHBE cells, the infected NHBE cells are not amenable for plunge freezing and subsequent cryo-EM studies. Therefore, we employed chemical fixation and immunogold labeling techniques, as we have done previously with RSV [44]. With this approach, we were able to obtain structural information from the RSV-infected polarized NHBE cells. In humans, the airway epithelial cells are the first line of defense in the respiratory tract and are the primary sites for RSV infection; therefore, understanding RSV structure and its assembly process from these polarized cells provides information regarding RSV pathogenesis and its associated diseases [62,63]. Differentiated NHBE cells are polarized cells that have been cultured on air-liquid interface platforms. RSV is known to mature and release from the apical surface of polarized epithelial cells [48,64,65,66]. NHBE cells have cellular filaments such as cilia and microvilli, which may make it difficult to differentiate RSV filaments from cell-based structures. However, RSV particles have a 10–20 nm layer of glycoproteins coating the viral membrane and a consistent diameter of ~130 nm (Figure 5A–C). Cilia have a clear “9 + 2” doublet by cross section and lack surface glycoproteins (Figure 5D,E) and microvilli are significantly smaller in diameter and have thin, filamentous external structures. The characteristic morphologies of the virus and cellular appendages allowed us to readily differentiate the RSV filaments from microvilli and cilia (Figure 5E). The TEM images demonstrate that RSV particles produced by and extending from the infected-NHBE cells are filamentous (Figure 5). In all, the results suggest that RSV morphology is filamentous in both transformed cell lines and polarized human airway epithelial cells.

### 3.5. The RSV F Glycoprotein Is in Prefusion form on Filamentous Particles and in Postfusion form on Spherical Particles

**Figure 6:**
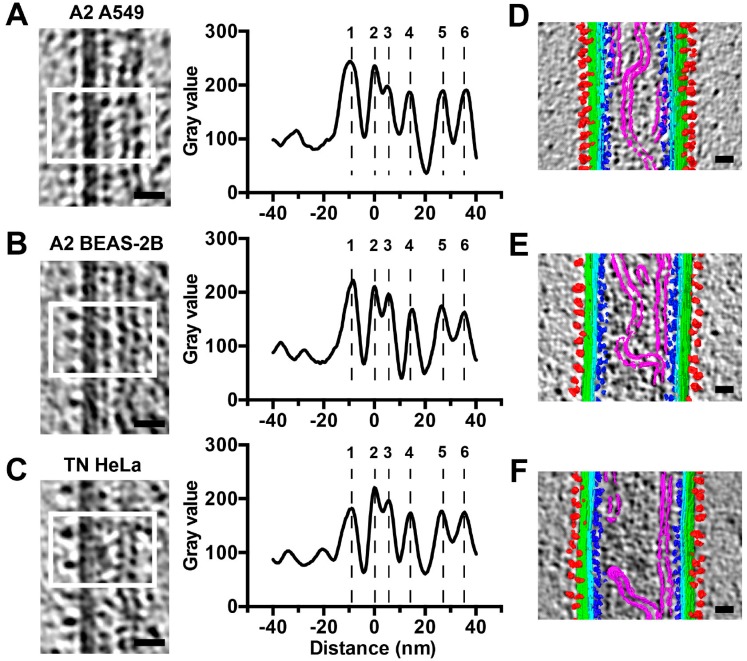
Structural analysis of RSV filamentous particles. (**A**–**C**) Representative tomographic slices and linear density profiles of RSV ultrastructure. White boxes indicate representative regions used to generate linear density plots. Linear density profiles are averaged results of multiple line profiles from several regions. (**D**–**F**) Segmentation illustration of the filamentous particles from indicated virus-infected cells. Note that the segmentation areas do not correspond to the regions in (**A**–**C**), but representative regions of samples from (**A**–**C**). Density peaks 1 through 6 represent: 1, Viral glycoproteins (Red); 2, Viral membrane (Green); 3, Matrix (Cyan); 4, M2-1 (Blue); 5 and 6, RNP (Magenta). Scale bars are 20 nm.

**Figure 7:**
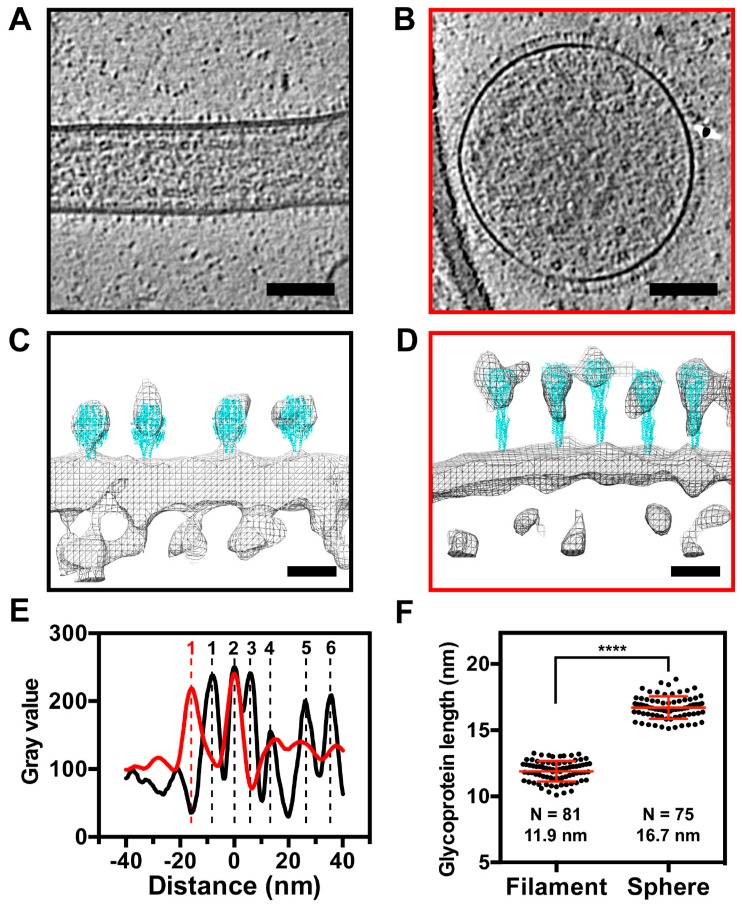
Structural comparison between filamentous and spherical RSV particles. (**A**,**B**) Representative tomographic slices of a filamentous (**A**, black outline) and spherical (**B**, red outline) particle. (**C**,**D**) Model fitting of representative regions of the isosurface rendering of the tomographic data for filamentous (**C**, black outline) and spherical (**D**, red outline) particles. Prefusion F (PDB ID: 4JHW) and postfusion F (PDB ID: 3RRT) were used for (**C**) and (**D**), respectively. (**E**) Linear density profiles of a filamentous particle (black line) and spherical particle (red line). Density peaks 1 through 6 represent: 1, Viral glycoproteins; 2, Viral membrane; 3, Matrix; 4, M2-1; 5 and 6, RNP. (**F**) Glycoprotein length quantification from filamentous and spherical particles. **** indicates the student *t*-test *p*-value is below 0.0001. Scale bars are 100 nm (**A**,**B**) and 10 nm (**C**,**D**).

Linear density profiles of the released virus particles show that both the A2 and TN strains have very similar spatial organization with respect to the RSV structural components (Figure 6). There is a surface glycoprotein peak ~10 nm outside of the viral membrane, with the M protein ~5 nm underneath the viral membrane [12]. The fact that the peak density of the glycoprotein layer is ~10 nm away from the viral membrane, indicates that the majority of RSV F are in the prefusion conformation [24]. This measurement is different from what was reported from the purified particles, where a mixture of the two major conformational states was observed [11,12]. The linker protein, M2-1, is present between M and RNPs, with a peak density ~12 nm away from the M protein. This was consistent with our previous study of purified RSV particles using Zernike Phase Contrast (ZPC) tomographic data [11]. The helical RNPs are located under the M2-1 layer, resulting in two peaks from the densities on either side of the helix (Figure 6A–C), which is in agreement with previous studies of purified filamentous particles [11,12]. In addition to the filamentous particles, ~5% of the imaged particles were spherical (Figure 7). We analyzed the structures of the glycoproteins and M protein layers of the filamentous and spherical particles by preparing linear density profiles and modeling the F glycoprotein crystal structures into EM-density maps (Figure 7). Linear density profiles and glycoprotein length quantification suggest that the glycoproteins on the filamentous particles are ~5 nm shorter than the glycoproteins on the spherical particles (Figure 7E,F). Modeling the crystal structures into the EM density suggests that the RSV F glycoproteins are in the metastable prefusion form on the filamentous particles, while they are in the stable postfusion form on the spherical virus particles (Figure 7C,D). Furthermore, there is a missing peak at the M density layer in the spherical particles, indicating that the M layer is detached from the membrane (Figure 7D,E). These results agree with our recent findings using sub-volume averaging analysis [24]. 

### 3.6. RSV Assembly Steps Revealed by Cryo-ET

**Figure 8:**
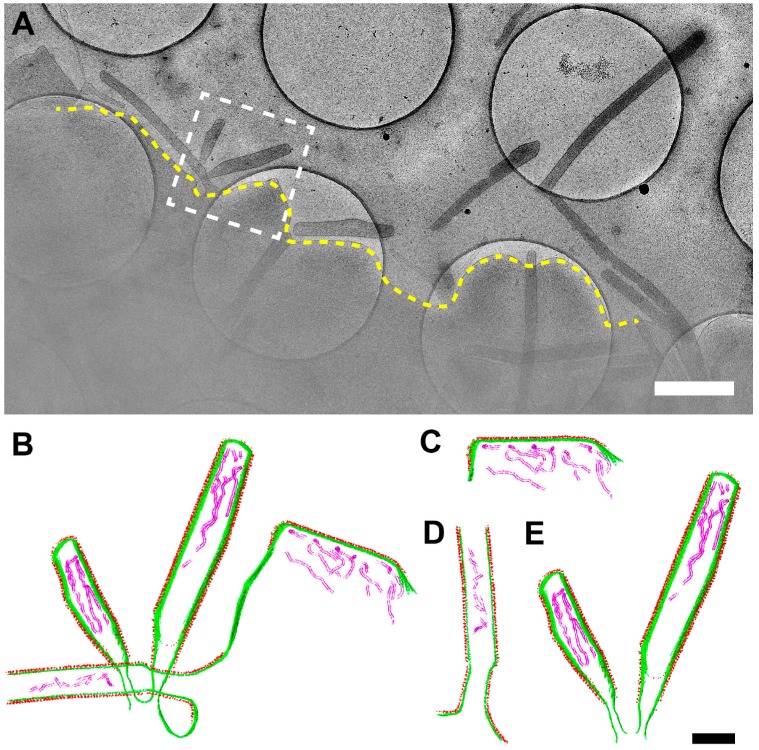
RSV assembly steps revealed by cryo-ET. (**A**) Representative cryo-EM images of RSV-infected cells. Note the assembly site indicated by the dashed white box and the released viral particles. The dashed yellow line indicates the cell plasma membrane. (**B**) Segmentation of the assembly site indicated in the white dashed box in (**A**), illustrating initiation (**C**), elongation (**D**), and scission (**E**) events. See also Supplementary Movie S1. Green is membrane, red is glycoprotein, and magenta is the RNP complex. Scale bars are 1 µm (**A**) and 200 nm (**B**–**E**).

We investigated the steps of RSV assembly from infected cells to understand how RSV forms as a filamentous particle. By collecting cryo-ET data at the sites of assembly, we were able to capture the different stages of RSV genesis: initiation, elongation, and scission (Figure 8, Supplementary Movie S1). Initiation is characterized by accumulation of viral components at the plasma membrane but precedes protrusion of the viral filament from the membrane (Figure 8C). Elongation occurs when the viral filament has protruded from the membrane and is actively extending (Figure 8D). Scission is defined as narrowing of the RSV filament diameter at the cell proximal end of the filament and is followed by the release of RSV particles from the cell (Figure 8E). It is clear that RSV develops as a filamentous particle, demonstrated by elongation, scission, and release steps.

**Figure 9:**
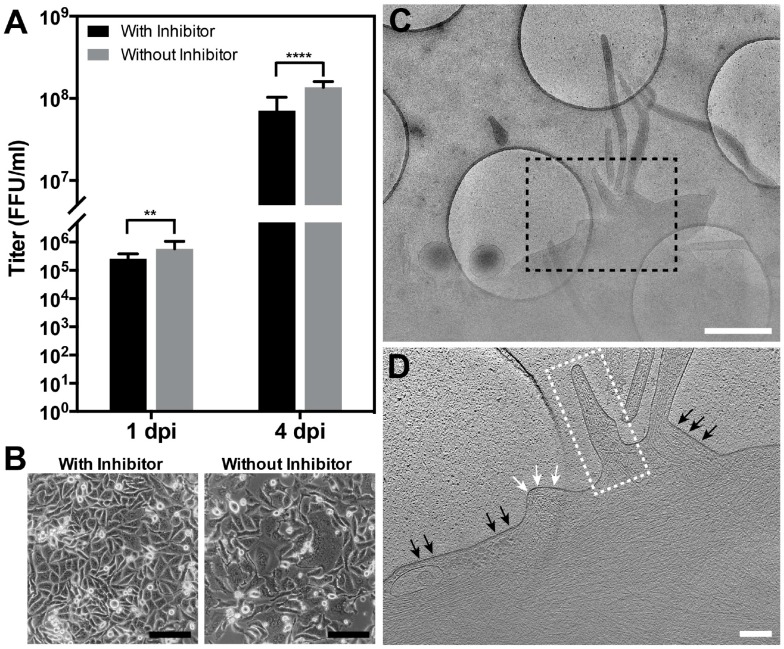
RSV assembly in the presence of fusion inhibitor. RSV-infected HeLa cells were infected at a M.O.I. of 10, washed twice with PBS prior to adding 600 nM RSV fusion inhibitor (BMS-433771), and incubated for the indicated length of time. (**A**) Titration results of RSV-infected cells with or without RSV fusion inhibitor at 1 and 4 d.p.i. ** indicates the student *t*-test *p*-value is below 0.01. **** indicates the student *t*-test *p*-value is below 0.0001. (**B**) Representative images showing the absence of syncytial formation with inhibitor and syncytial formation without inhibitor. (**C**) Polygon montage of an assembly site. Micrographs were acquired using cryo-EM/ET, demonstrating the assembly events at 24 h.p.i. (**D**) Tomographic slice (6.14 nm) of the assembly site collected from the region indicated by the dashed black box in (**C**). The black arrows indicate the early assembly events prior to RSV filament elongation. Note the presence of matrix layer and the glycoproteins. The white arrows indicate the plasma membrane; note the absence of the matrix layer and the glycoproteins. The dashed white box indicates an elongating RSV filament. Scale bars are 100 μm (**B**), 1 μm (**C**), and 200 nm (**D**).

In order to validate and distinguish assembly from entry and fusion events, we incubated the infected cells with the fusion inhibitor, BMS-433771. BMS-433771 is a small-molecule inhibitor that can block RSV fusion [51,52,67,68]. Mechanistically, the fusion inhibitor binds to the hydrophobic binding pocket of the prefusion RSV F [52,68], resulting in the inhibition of RSV-induced syncytium formation and decreased viral titers [67]. Our titration results showed statistically significant differences between groups with and without fusion inhibitor treatment (Figure 9A). In addition, by examining syncytium formation, we noticed that in the presence of the fusion inhibitor, the infected cells did not form syncytia while the control group did (Figure 9B), validating the result that the fusion inhibitor decreases viral titers. Our cryo-ET analysis demonstrated that assembly events were still resolved (Figure 9C,D) and were very similar to those processes seen when RSV and cells were cultured in the absence of the fusion inhibitor (Figure 8, Supplementary Movie S1). This confirms that RSV assembles as filamentous particles from infected cells, and that the events imaged in these experiments were assembly events rather than entry events.

## 4. Discussion

To our knowledge, this is the first study to determine the structures associated with RSV particle assembly from infected human cell lines and polarized NHBE cells by multiple EM imaging modalities. The collective results reveal that RSV assembly occurs at the plasma membrane (Figure 8 and Figure 9, Supplementary Movie S1) and the native-state RSV particles are filamentous (Figure 2, Figure 3 and Figure 5). Our results differ from a recent report from the Santangelo group. Using fluorescence microscopy on RSV-infected cells, they demonstrated that RSV may pre-assemble as filaments in the cytoplasm prior to particle formation or elongation at the plasma membrane [43]. Our study focused on events localized at the plasma membrane due to sample thickness limitations that precluded investigations of regions thicker than 500 nm. Therefore, this alternative assembly pathway is un-tested by cryo-ET. However, future studies could utilize cryo-focused ion beam scanning electron microscopy (cryo-FIB-SEM) and correlative light and electron microscopy (CLEM) methodologies to investigate deep cytoplasmic events, thereby enhancing our understanding of RSV assembly.

We have established an optimized system for imaging RSV-infected cells on TEM grids by cryo-EM and cryo-ET. In order to balance the low-density of cells and the high-quantity of viral filaments, we selected a M.O.I. of 10 and cryo-preserved the samples at 24 h.p.i., which was comparable to other closely related paramyxovirus studies [69]. To further validate the cryo-sample preparation conditions, we carried out a series of analyses including virus titration, real time RT-PCR assays, and flow cytometry to monitor the virus growth dynamics, viral RNA quantification, and fluorescent protein production. Our results show that at a M.O.I. of 10 and 24 h.p.i., RSV-infected cells actively produce a large quantity of infectious particles, as indicated by the significantly higher viral titer comparing to the other conditions at lower M.O.I. (Figure 1). The formation of filamentous RSV particles using these conditions has been validated by both fluorescence light microscopy and cryo-ET [24,44,45,59].

Three distinct morphologies were previously categorized from purified RSV particle preparations by cryo-ET: filamentous, spherical, and a structural intermediate [11,12]. Here, using whole cell cryo-ET imaging, we report that RSV is filamentous when newly released from infected cells. Consistent with the cryo-EM/ET findings, conventional immunogold TEM demonstrates that assembling and released RSV particles from RSV-infected HeLa cells are filamentous [44]. It has been suggested that the morphology of influenza virions is dependent on the phenotype of polarized cells and the integrity of the cytoskeleton [70]. Specifically, A/Udorn/72 viruses produced from infected polarized epithelial cells were filamentous and when non-polarized cell types were infected by A/Udorn/72 viruses, the virions were almost exclusively spherical [70]. It was recently demonstrated that infection of human bronchial epithelial cells by human metapneumovirus (HMPV) leads to the formation of branched cellular networks and cell-associated filaments. This indicates that the formation of the filamentous networks is important for cell-to-cell transmission of the viral particles [71]. By investigating the structure of two RSV strains produced in multiple non-polarized cell lines and polarized NHBE cells by cryo-EM, cryo-ET, and conventional TEM, we conclude that RSV is filamentous independent of virus strain, cell line, and polarization phenotype of the cells (Figure 3 and Figure 5). 

**Figure 10:**
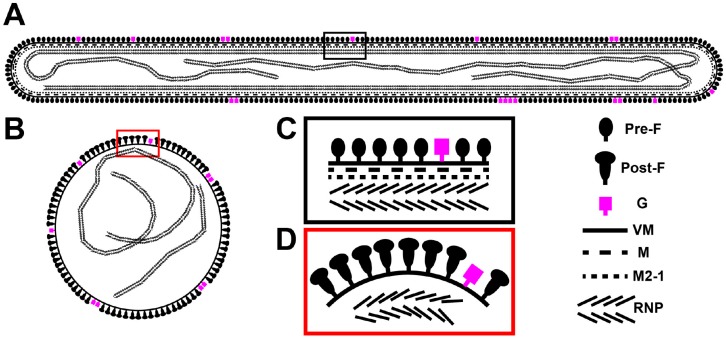
RSV morphology model. A schematic representation of a filamentous (**A**) and a spherical (**B**) RSV viral particle. Enlarged views of the boxed regions in (**A**) and (**B**) are shown in (**C**) and (**D**), respectively. The structure of G and the ratio of G to F are unknown. In the filamentous particle (**A**,**C**), F is in the prefusion form with matrix (M) lining the viral membrane (VM). M2-1 acts as a linker protein between M and the RNP. In the spherical particle (**B**,**D**), F is in the postfusion form while M is detached from the viral membrane. RNP is disordered and not linked by M2-1 to the matrix protein.

In our study and previous reports on RSV structure by cryo-EM, we considered at length the function of the spherical virus particle, its infectivity, and at which stage it might form. We offer two possible hypotheses about spherical RSV particles. First, spherical RSV particles may be the result of morphological rearrangements of the filamentous particles, brought about by particle disruption, damage, or other events. This view is supported by the direct observation that RSV particles only bud as filaments (Figure 2, Figure 8 and Figure 9) [24,44,45,59]. We captured different stages of RSV assembly and demonstrated that the greatest percentage of particles was filamentous (Figure 8 and Figure 9), which suggested that the spherical particles result from the structural reorganization of the filamentous particles (Figure 7 and Figure 10). Alternatively, RSV may bud as spherical particles, similar to other enveloped RNA viruses. It has been shown that after prolonged infection, Marburg virus, a closely related filovirus, assembles and releases as spherical particles. However, this is accompanied by increased vesiculation of the plasma membrane and decreased infectivity of the released viruses [72,73]. We have shown that at 6 d.p.i., filamentous RSV particles are present on RSV-infected polarized NHBE cells (Figure 5), indicating that the second explanation is less likely for RSV morphogenesis. 

Structural comparisons between the filamentous and spherical RSV virions revealed significant differences associated with the surface glycoproteins (Figure 7 and Figure 10). The purified RSV particles that were reported by the Butcher group [12] illustrated that there was a mixture of the prefusion and postfusion forms of RSV F on filamentous virions. Our whole-cell cryo-ET studies of RSV-infected cells indicate that the filamentous RSV particles were covered with prefusion F and the spherical particles had predominantly the postfusion form, which was further supported by model fitting (Figure 7C,D), linear density profiles (Figure 7E), length measurements (Figure 7F), and sub-volume averaging analysis [24]. This provides a molecular mechanism that relates RSV structure and function, i.e., the relationship between RSV morphology and infectivity. During the RSV entry process, the fusion of the viral membrane with the cellular membrane is facilitated by the RSV F fusion peptide [74]. The fusion peptide is exposed when the RSV F glycoprotein undergoes a conformational change from the prefusion to the postfusion form. Thus, the maintenance of the prefusion form on RSV particles is required for RSV infectivity. Based on the structural analysis of these two morphologies and other published results [12], filamentous RSV is the infectious form of the virus. In support of this model, it has also been demonstrated in Marburg virus that the infectious particles are filamentous, as revealed by infectivity assays and electron tomography of plastic embedded infected-cells [72].

In summary, our study explored RSV morphology in transformed cell lines as well as in polarized human airway epithelial cells, and we conclude that RSV morphology is largely filamentous from infected-cells independent of cell lines or virus strains. Structural analysis of RSV-infected cells indicates that in the filamentous particles, RSV F is predominantly in the metastable prefusion conformation with M underlying the viral membrane and the RNP linked to M by M2-1. In the spherical particles, RSV F is mainly in the stable postfusion form and M and M2-1 are detached from the viral membrane (Figure 10), suggesting that filamentous RSV is more infectious. Finally, RSV assembles at the plasma membrane and buds off as filaments, providing a morphogenesis mechanism for filamentous RSV particles. This study expands the existing knowledge of RSV architecture by offering morphological and structural analyses of RSV in the native state. 

## Supplementary Materials

The following are available online at http://www.mdpi.com/1999-4915/10/8/446/s1, Supplementary Movie S1. Cryo-ET of RSV assembly. Tomographic reconstruction and corresponding segmentation of a RSV assembly site captured by cryo-ET, revealing the initiation, elongation, and scission stages. The color scheme is the same as Figure 8. Green is membrane, red is glycoprotein, and magenta is the RNP complex.
